# Injury-Dependent and Disability-Specific Lumbar Spinal Gene Regulation following Sciatic Nerve Injury in the Rat

**DOI:** 10.1371/journal.pone.0124755

**Published:** 2015-04-23

**Authors:** Paul J. Austin, Alison L. Bembrick, Gareth S. Denyer, Kevin A. Keay

**Affiliations:** 1 School of Medical Sciences (Anatomy & Histology), The University of Sydney, Sydney, NSW, Australia; 2 School of Molecular Bioscience, The University of Sydney, Sydney, NSW, Australia; University of Cincinnati, UNITED STATES

## Abstract

Allodynia, hyperalgesia and spontaneous pain are cardinal sensory signs of neuropathic pain. Clinically, many neuropathic pain patients experience affective-motivational state changes, including reduced familial and social interactions, decreased motivation, anhedonia and depression which are severely debilitating. In earlier studies we have shown that sciatic nerve chronic constriction injury (CCI) disrupts social interactions, sleep-wake-cycle and endocrine function in one third of rats, a subgroup reliably identified six days after injury. CCI consistently produces allodynia and hyperalgesia, the intensity of which was unrelated either to the altered social interactions, sleep-wake-cycle or endocrine changes. This decoupling of the sensory consequences of nerve injury from the affective-motivational changes is reported in both animal experiments and human clinical data. The sensory changes triggered by CCI are mediated primarily by functional changes in the lumbar dorsal horn, however, whether lumbar spinal changes may drive different affective-motivational states has never been considered. In these studies, we used microarrays to identify the unique transcriptomes of rats with altered social behaviours following sciatic CCI to determine whether specific patterns of lumbar spinal adaptations characterised this subgroup. Rats underwent CCI and on the basis of reductions in dominance behaviour in resident-intruder social interactions were categorised as having *Pain & Disability*, *Pain & Transient Disability* or *Pain alone*. We examined the lumbar spinal transcriptomes two and six days after CCI. Fifty-four ‘disability-specific’ genes were identified. Sixty-five percent were unique to *Pain & Disability* rats, two-thirds of which were associated with neurotransmission, inflammation and/or cellular stress. In contrast, 40% of genes differentially regulated in rats without disabilities were involved with more general homeostatic processes (cellular structure, transcription or translation). We suggest that these patterns of gene expression lead to either the expression of disability, or to resilience and recovery, by modifying local spinal circuitry at the origin of ascending supraspinal pathways.

## Introduction

The affective-motivational consequences of nerve injury have become an emerging focus of rat models of neuropathic pain. The negative affective consequences of the spared nerve injury (SNI) [1]; spinal nerve ligation (SNL) [2]; and chronic constriction injury of sciatic nerve (CCI) [3] models of neuropathy, have been assessed using conditioned place aversion / place escape avoidance paradigms, and inferred from analgesic conditioned place preference [4–8]. These studies provide evidence for the immediate (day-3 post injury) and persisting (4 weeks post-injury) perception of unpleasantness of neuropathic injury [4, 5, 8], and for its reversal by: (i) analgesics acting at spinal alpha-2 adrenoreceptors or, N-type calcium channels and; (ii) inhibition of supra-spinal facilitatory pathways [6, 7].

In tests, which evaluate anxiety-like and depression-like behaviours, SNL appears to have little effect [9], whereas, CCI triggers clear anxiety and depression-like behaviours in all injured rats [10–16]. Further in the SNI model, rats show anxiety-like behaviours [17, 18]. Anxiety and depression-like behaviours have been first reported at 7 days following injury and persist for up to 6 months after injury.

Attempts to evaluate the impact of nerve injury on hedonic processes have resulted in equivocal findings. The continuous monitoring of sucrose (15%) preference in individual rats reveals an anhedonic impact of CCI from day 7 post-injury onwards [19]. In contrast, episodic sucrose preference testing revealed no effect (2 consecutive nights, week 3 post-injury), similarly, the ingestion of sweet cereal rewards at two weeks post-injury was not impacted by CCI [5, 15].

Sleep-wake cycles are also disrupted by nerve injury (although see [20]). SNI increased the frequency of episodes of wakefulness and slow-wave sleep [21]. CCI also disrupts the sleep-wake cycle, however, the precise characteristics of the disruptions appear to depend on the strain of the rat, the diurnal phase of measurement, the housing conditions, whether one or two nerves are ligated, as well as the post-injury time of recordings [22–24].

On balance, of the studies investigating affective-motivational changes following nerve injury, the CCI model has been the most widely investigated and has revealed the most robust findings. Specifically, by the first week following injury, CCI triggers a state of unpleasantness, associated with anxiety and depression-like behaviours, coupled to varying degrees of anhedonia and sleep disturbance.

Work from our laboratory has focused on the CCI model and has shown changes in social interactions [25–30], sleep-wake-cycle [27] and endocrine function (hypothalamo-pituitary adrenal & thyroid) [31, 32] immediately after the injury in half of CCI rats. We have shown that these changes persist for at least sixteen days in a subgroup of approximately one third of CCI rats and that this subgroup of rats can be reliably identified after only six days of observation [27]. The subgroup was originally identified from observations in a large cohort of CCI rats (n = 46), which leads us to suggest that in studies with smaller cohorts, this subpopulation may simply be regarded as outliers. CCI consistently produced thermal and mechanical, allodynia and hyperalgesia, the intensity of which was unrelated to the presence of altered changes in social interactions [25–30], sleep-wake-cycle [27] and endocrine function [31, 32]. A decoupling of the sensory consequences of nerve injury (allodynia and hyperalgesia) from the affective-motivational changes triggered by nerve injury have also been reported in the place escape avoidance paradigm following lesions or direct stimulation of the anterior cingulate cortex [33, 34]; conditioned place aversion following low dose morphine administration [8] and; conditioned place preference following either intra-thecal adenosine injections or lesions of the anterior cingulate [6, 35]. In humans a similar dissociation of sensation and affective-motivational state is illustrated by: (i) ‘non-patients with chronic pain’, individuals with chronic pain who do not seek medical treatment, these people are often missing in clinical reports, although have been used as controls for clinical chronic pain patients in some studies [36–39] and; (ii) the poor concordance of pain intensity with disruption of quality of life [40–44].

This decoupling of the sensory versus affective-motivational consequences of nerve injury described in rats and humans is broadly accepted to reflect that each are represented at anatomically distinct supra-spinal sites. Furthermore, these observations suggest that affective-motivational state changes are also driven, by some, but not all elements of the spinal circuits that trigger sensory changes [6, 45].

Neural circuits in the sciatic nerve recipient dorsal horn of L4 to L6 lumbar spinal segments primarily mediate the sensory changes triggered by sciatic nerve CCI. Consequently, this region has been the focus of transcriptome studies whose aim has been to identify nerve injury-evoked gene expression, which may lead to neuropathic pain [46–49]. The critical role of these transcriptional changes in the spinal adaptations specific to either sensory or, affective-motivational changes cannot be determined in these studies. The fact that we can define subgroups of rats either with, or without, altered social behaviours and sleep-wake cycles after CCI, does permit the identification of specific transcriptional changes that may play important roles in the expression of certain disrupted behaviours. In these studies, we used microarrays to identify the unique transcriptomes of rats with altered social behaviours following sciatic CCI to determine whether specific patterns of spinal adaptations characterised this subgroup. We examined the spinal transcriptomes two days and six days after CCI and in line with a recent meta-analysis of microarray studies [49], our analysis focused on the key processes of; (i) neurotransmission, (ii) inflammation and/or cellular stress, (iii) cellular signalling; (iv) cellular structure; (v) ionic balance and (vi) transcriptional and translational processes. Because our overall aim was to describe the spinal adaptations specific to altered activity in spinal neurons that might drive affective-motivational change, our first step was to interrogate the transcriptional changes in the neurotransmission functional group using RT-PCR.

## Methods

### Animals

All experimental procedures were carried out in accordance with: the guidelines of the NHMRC “Code for the Care and Use of Animals in Research in Australia”; the “Ethical Guidelines for Investigations of Experimental Pain in Conscious Animals” laid down by the International Association for the Study of Pain [50] and; the ARRIVE guidelines (https://www.nc3rs.org.uk/arrive-guidelines). Furthermore, the University of Sydney Animal Care and Ethics Committee approved all procedures (# 3920). All procedures were designed to minimise the intensity and duration of animal suffering as well as animal numbers, within the context of addressing the experimental aims.

Experiments were performed on outbred, male Sprague-Dawley rats, (ARC, Australia) weighing 250g-310g on the day of CCI. Male rats were selected to remove the need to control for oestrus cycle related variation in spinal cord gene regulation. Rats were housed individually in clear Perspex cages in an animal house maintained on a reversed 12/12 light/dark cycle with lights on at 19–30h, with food and water available *ad libitum*. The behavioural analyses were conducted during the dark phase of the circadian cycle. Room temperature was maintained at 22 (±1)°C.

### ‘Resident-intruder’ social interactions testing

Each ‘resident’ rat was habituated to its home-cage for a period of one-week, before the introduction of a novel, sex, age and weight-matched conspecific of the same strain, (the ‘intruder’) for six minutes every day (see [27] for complete details). Rats were allocated randomly to either the ‘resident’ or the ‘intruder’ groups. Briefly, resident-intruder social interactions (6 minutes) were analysed for five days pre-CCI. The CCI surgery was performed on the day after social interactions testing on day five. Post-CCI behavioural testing continued for either two days (n = 27), or six days (n = 88).

Testing was performed at 09–30h each day 2 hours after lights off. Using an infrared camera each rat was recorded for one minute prior to the introduction of the intruder, and then for six minutes in the presence of the intruder. Resident rats never encountered the same intruder on consecutive days, and never more than twice throughout testing.

### Chronic constriction injury of the sciatic nerve

CCI was performed identical to that first described by Bennett and Xie [3]. Briefly, anaesthesia was induced with 5% halothane in 100% O_2_ (Laser Animal Health, Australia), and maintained via a custom made facemask (2% in 100% O_2_). The ability to control the depth of anesthesia and the rapid trajectory of recovery were the reasons that halothane anesthesia was chosen. The right sciatic nerve was exposed by blunt dissection through the biceps femoris and four ligatures (chromic gut, 5.0, Ethicon, Somerville, NJ, USA) were loosely tied, 1mm apart, just proximal to the trifurcation of the sciatic nerve. Constriction was minimal to cause “visible retardation, but not arrest, of the epineural vasculature” as originally defined [3]. The incision was sutured (Mersilk, 5.0, Ethicon) and iodine solution (Povidone-Iodine, Orion Laboratories Pty. Ltd., Australia) and triple antibiotic powder (Tricin, Sigma) were applied topically. Each rat was observed closely during its recovery period and during the 24h following its return to the home-cage.

### Behavioural analyses

The residents behaviour during the six minute test period was quantified within four mutually exclusive categories:

- **Dominance behaviour:** standing on top of the supine intruder, back or lateral attack with biting targeted at the neck or back of the intruder, and chasing the intruder.
- **Social behaviour:** investigation or sniffing of the intruder with particular focus on anogenital region.
- **Non-social behaviour:** cage exploration and self-grooming.
- **Submissive behaviour:** defensive alerting/freezing, defensive sideway or supine posture upon the approach of the intruder.

These categories were identical to those used previously by Monassi and colleagues, and are based on earlier descriptions of Grant and Mackintosh [51].

Development of stable resident-intruder interactions requires prior intruder exposure therefore the behaviour of each resident on the post-CCI days was compared with its behaviour on the 3 days immediately prior to CCI (i.e. pre-CCI days 3–5). Resident’s were then categorised based upon changes in the duration of dominance behaviour post-CCI as follows; for rats tested for six days post-CCI, we identified the three subgroups defined in our earlier studies: (i) *Pain alone*: No change in the duration of dominance behaviour post-CCI, compared to pre-CCI. (ii) *Pain & Disability*: A reduction of at least 30% in the duration of dominance behaviour on at least 5 out of 6 post-CCI days, compared to pre-CCI days. (iii) *Pain & Transient Disability*: An initial transient reduction of at least 30% in the duration of dominance behaviour on the first 3–4 days post-CCI, compared to pre-CCI, followed by a return to pre-CCI levels (days 4/5-6).

For rats tested for two days post CCI, only **two** subgroups were selected for analysis: (i) rats that showed no change in the duration of dominance behaviour on post-CCI days 1 and 2, which are predicted to become *Pain alone*; and (ii) rats that showed a greater than 30% decrease in dominance on each of post CCI days 1 and 2, which are predicted to be either *Pain & Disability* or *Pain & Transient Disability*.

Mechanical and thermal threshold testing alters gene expression [52, 53] and because we have previously reported that changes in allodynia, hyperalgesia and spontaneous pain behaviours occur in all sciatic nerve CCI animals [25, 27], it was not conducted on these rats.

### Statistical analysis of behaviour

A two way between-groups ANOVA evaluated the effects of time and post-injury behavioural group on dominant, social, non-social and submissive behaviours. Significant main effects were probed further using Bonferroni *post-hoc* comparisons between each of the behavioural groups. The results are presented as means (± SEM).

### Sacrifice

Following ‘resident-intruder’ testing each animal was rapidly decapitated under CO_2_ narcosis. The spinal cord was immediately exposed and whole spinal cord segments L4-L6 were isolated and the dura and rootlets were removed, before being placed in 0.2ml ice cold TRI-reagent (Sigma, Castle Hill, NSW, Australia). Whole spinal cords were analysed because by days 2 and 6 post-CCI, bilateral changes in spinal cord gene regulation have been reported in other studies [54–57]. These spinal cord blocks were then stored at -80°C until processed further. In addition, twelve uninjured rats served as controls, six were used for microarray studies and six for RT-PCR experiments.

### Microarray studies

#### Day 2 post-CCI

two pooled samples of RNA, each containing spinal cords from three rats from each of the *Pain alone (day 2)*; *Pain & Disability* / *Transient Disability (day 2)* and uninjured control groups were analysed (6 microarrays).

#### Day 6 post-CCI

On two independent occasions, pooled samples of RNA extracted from spinal cords of three rats from the *Pain alone (day 6)*; *Pain & Disability (day 6); Pain & Transient Disability (day 6)* and uninjured control groups respectively, were analysed (4 microarrays per independent experiment).

Replicate pooled samples were chosen for analysis in this study to highlight genes most likely to characterise rats with or without disabilities, an approach identical to that used in other microarray studies [29, 46, 47]. Pooled samples also strengthen the analysis offered by the Affymetrix Microarray Analysis Suite (MAS) version 5.0.

Total RNA was extracted from the spinal cord samples using the manufacturer’s TRI-reagent protocol (chloroform-phenol method). Total RNA was then purified using the GenElute mammalian Total RNA kit (Sigma). RNA was assayed at wavelengths of 260 nm, 280 nm and 320nm using a UV spectrophotometer (Biochrom Ltd, UK) to determine the RNA concentration and 260nm/280nm ratio. At this point 6.67μg of RNA from the three spinal cord samples designated for each group were pooled together, giving a total of 20μg per group. Using 20 μg of total RNA, double stranded cDNA was synthesised for each sample in a two-step reaction, using a “SuperScript Double-Stranded cDNA Synthesis Kit” (Invitrogen, Mulgrave, VIC, Australia). The cDNA was then purified using a phenol/chloroform extraction with Phase-lock gels (Eppendorf, Wesseling-Berzdorf, Germany), and then resuspended in RNase free water. This step was followed by *in vitro* transcription labelling with biotinylated-UTP and-CTP, performed according to the manufacturer's recommendations to produce cRNA (Enzo Diagnostics; Millenium Science, Mulgrave, VIC, Australia). The cRNA transcripts were then purified, using a GenElute mammalian Total RNA kit (Sigma) and then quantitated and assayed, again using the UV spectrophotometer. The cRNA was fragmented and the suitability of the samples for further analyses was confirmed using Affymetrix GeneChip Test3 arrays (low background, good overall signal intensity and low 3’/5’ ratios of housekeeping genes). Affymetrix Test-3 chips showed that all samples met each of the criteria required for further analysis using the Rat Neurobiology U34 GeneChip (RNU34). Each sample was subsequently hybridised for 16 hours to the RNU34 microarray following the standard Affymetrix protocol. The arrays were then washed and stained according to the Affymetrix fluidics station Mini_euk2v3 protocol, followed by scanning in a gene array scanner (Hewlett Packard Instruments, TX, USA).

### Microarray data analysis

Affymetrix Microarray Analysis Suite (MAS) version 5.0 was used to analyse the microarrays using Wilcoxon signed-rank test to generate a statistically significant signal detection value and then; (i) determine the presence, and quantify the level of expression, of transcripts within each probe set and; (ii) calculate the magnitude and call the significance of the fold change. Genes of interest were identified, using a custom designed analysis tool [58], which identified increased (‘present’ call in sample) or decreased (‘present’ call in control) regulation from greater than, or equal to, 1.3 fold compared to uninjured controls. On day 2 post-CCI, because all microarrays were run at the same time, four pairwise comparisons were possible between control and behavioural groups, therefore only two identical ‘present’ calls were required for a gene of interest to be identified. In contrast, because post-CCI day 6 comparisons were conducted on two separate occasions, only two pairwise comparisons were made between control and behavioural groups, therefore genes of interest were identified by two identical present calls. In addition, day 2 biological replicates were compared to each other to determine the degree of technical and biological variation between the samples. Genes were subsequently categorised, at each time point, as either (i) genes similarly regulated in all rats in response to nerve injury, that is ‘injury-dependent’ genes, which may be involved in sensory-discriminative aspects of pain, or (ii) genes uniquely regulated with respect to changes in social behaviours, that may be important in driving complex behavioural change following nerve injury, that is ‘disability-specific’ genes. It is important to note that, at day 2 post-CCI *Pain & Disability* and *Pain & Transient Disability* rats cannot be distinguished from each other, and constitute the *Pain & Disability* / *Transient Disability (day 2)* group. Genes were then grouped according to their protein-encoding function.

### Real-time RT-PCR

Real-time RT-PCR was conducted for 17 selected genes from the neurotransmission group of transcripts compared to ribosomal 18S as the housekeeping gene. The level of expression of ribosomal 18S RNA remains stable under different experimental conditions or treatments, unlike beta-actin, GAPDH and ubiquitin [59, 60]. Moreover, in our microarrays GAPDH was differentially regulated, whilst 18S the housekeeping gene chosen for our PCR was confirmed as having similar cycle threshold (Ct) values in each sample for the same amount of total RNA.

The primers used (listed in **Table 1**) were designed using the Primer Express/Gene Jockey programs to determine the Tm and to check for secondary structures and primer dimers. Primers were designed so that, where possible, the amplicon would cross at least one exon/intron boundary. All primers were custom made (Sigma Genosys, Castle Hill, NSW, Australia). In addition to analysis of the two replicates from the microarray experiments, RNA was extracted from a further three rats for each behavioural group to provide n = 3 replicates from 9 individual rats. The uninjured control samples were run in triplicate and contained equal amounts of RNA from six spinal cords pooled. Using 5μg total RNA for each sample, double stranded cDNA was synthesised using SuperScript III (Invitrogen). The cDNA synthesised was subjected to real-time PCR in the presence of SYBR Green (1:31250, Life Technologies) and forward and reverse primers (see **Table 1**). Amplification was performed with an initial denature at 95°C for 120s and then 30–40 cycles of 95°C for 30s (denaturing), 60–72°C for 30s (amplification) and 72°C for 30s (fluorescence data acquisition). A melt and gel analysis on resulting PCR products determined the presence of a single specific product. All PCR products including the housekeeping gene, 18S, were sequenced (n = 18), and confirmed the presence of a single matching PCR product for each primer set (SUPAMAC, Sydney NSW, Australia). In addition, no template controls (NTC) were run to determine the level of primer dimer formation and/or contamination and negative reverse transcriptase samples were run with the GABBR1 primers to confirm the absence of genomic contamination. The threshold cycle values were chosen in the linear range, and relative expression changes normalised to the housekeeping gene. Amplification efficiency was determined using the Rotor Gene v6 software. The ratio of expression of each gene in behavioural versus control samples was calculated using REST© (relative expression software tool) [61], which takes into account any variations in amplification efficiency. Statistics were performed in REST using the Pair Wise Fixed Reallocation Randomisation Test.

**Table 1:** Primer sequences for the genes examined using real time PCR.

Accession #	Gene	Primer Sequence	Length
M11188	Ribosomal 18S	Fwd 5′ GCCGCTAGAGGTGAAATTCTTG 3′	73 bp
		Rev 5′ GAAAACATTCTTGGCAAATGCTTT 3′	
AF020756	P2X2-3 Receptor	Fwd 5′ TGACAAGGGCAACATTGCAAGC 3′	290 bp
		Rev 5′ TGGCAAACCTGAAGTTGTAGCCTG 3′	
AI229237	Opioid receptor-like	Fwd 5′ AAGCCCAGGTCTCTCTGACACAG 3′	144 bp
		Rev 5′ TGTAACGCCCTTTCTATGGGTCAG 3′	
X55812	Cannabinoid receptor 1	Fwd 5′ TACCTGATGTTCTGGATTGGGGTG 3′	140 bp
		Rev 5′ GTGGATGATGATGCTCTTCTGGGTC 3′	
M93257	Catecholamine-O-methyltransferase	Fwd 5′ GCCAAAATAACAGCAGAGGCTCAG 3′	198 bp
		Rev 5′ CCCTGGCTGTCTTGGAACTCAC 3′	
D00688	Monoamine oxidase A	Fwd 5′ AAGGGTGATTCGCCAGCCAG 3′	195 bp
		Rev 5′ AATGGCTGGAACATCCTTGGACTC 3′	
AB016161	GABA-B1 Receptor	Fwd 5′ GGATGTGGAACCTTATTGTGCTCTTCA 3′	178 bp
		Rev 5′ GGTGGTCTGTTGGATGGTAGCGAT 3′	
AA852004	Glutamine synthetase	Fwd 5′ GCCTTCTAATGGCTTCCCTGGAC 3′	146 bp
		Rev 5′ ACCTCGGCATTTGTCCCTGTG 3′	
L08228	NMDA 1 subunit	Fwd 5′ GGTTCGGTATCAGGAATGCGACTC 3′	166 bp
		Rev 5′ GCTTCCTACGGGCATCCTTGTG 3′	
U08259	NMDA-2C subunit	Fwd 5′ CACCATTGGGTCTGGCAAAGTC 3′	191 bp
		Rev 5′ TTGCTGCTCATCACCTCATTCTTCTC 3′	
U08260	NMDA-2D subunit	Fwd 5′ GCTGTCTGGGTGATGATGTTCGTC 3′	150 bp
		Rev 5′ TGGATTTCCCAATGGTGAAGGTAGAG 3′	
J03624	Galanin	Fwd 5′ GGGATGCCAACAAAGGAGAAGAGAG 3′	134 bp
		Rev 5′ CAGTGGTAACTCCCTCTTGCCTGTG 3′	
M15191	Tachykinin	Fwd 5′ CAGAGGAAATCGGTGCCAACG 3′	125 bp
		Rev 5′ TGGGTCTTCGGGCGATTCTC 3′	
M15580	Neuropeptide Y	Fwd 5′ CTGTGTGGACTGACCCTCGCTC 3′	124 bp
		Rev 5′ GGTGATGAGATTGATGTAGTGTCGCAG 3′	
K02248	Somatostatin	Fwd 5′ GGACCCCAGACTCCGTCAGTTTC 3′	127 bp
		Rev 5′ CCAGGGCATCGTTCTCTGTCTG 3′	
X53944	Dopamine 3 Receptor	Fwd 5′ CTTGGAGGTGACAGGT GGAGTCTG 3′	165 bp
		Rev 5′ GGTGCCGTGCTGATAGTGAACTG 3′	
J05122	Peripheral Benzodiazepine Receptor	Fwd 5′ TGGTATGCTAGCTTGCAGAAACC 3′	85 bp
		Rev 5′ CGAATACAGTGTGCCCCAGAT 3′	
M58587	Interleukin-6 receptor	Fwd 5′ GAATGGACTACCACGGGAAACACAC 3′	202 bp
		Rev 5′ GTGCTGCTTGGATGCCACTCAC 3′	

## Results

### Resident-Intruder Social Interactions testing

Resident-intruder testing was carried out for 5 days prior to CCI during which time a stable pattern of baseline behaviour was established. For the first minute after the introduction of the intruder, the resident displayed predominantly social behaviours. Following this, the resident began to display dominance behaviours towards the intruder, particularly, chasing, followed often by lateral and back attacks with biting targeted at the neck or back of the intruder, who characteristically responded with submissive behaviour. Towards the end of the 6-minute period the resident increasingly spent more time engaged in non-social and social behaviours. Submissive behaviour was rarely displayed. On pre-CCI days 3–5 dominance behaviours accounted for around 40% of the 6-minute test period.

#### Post CCI Day 2

**Fig 1:**
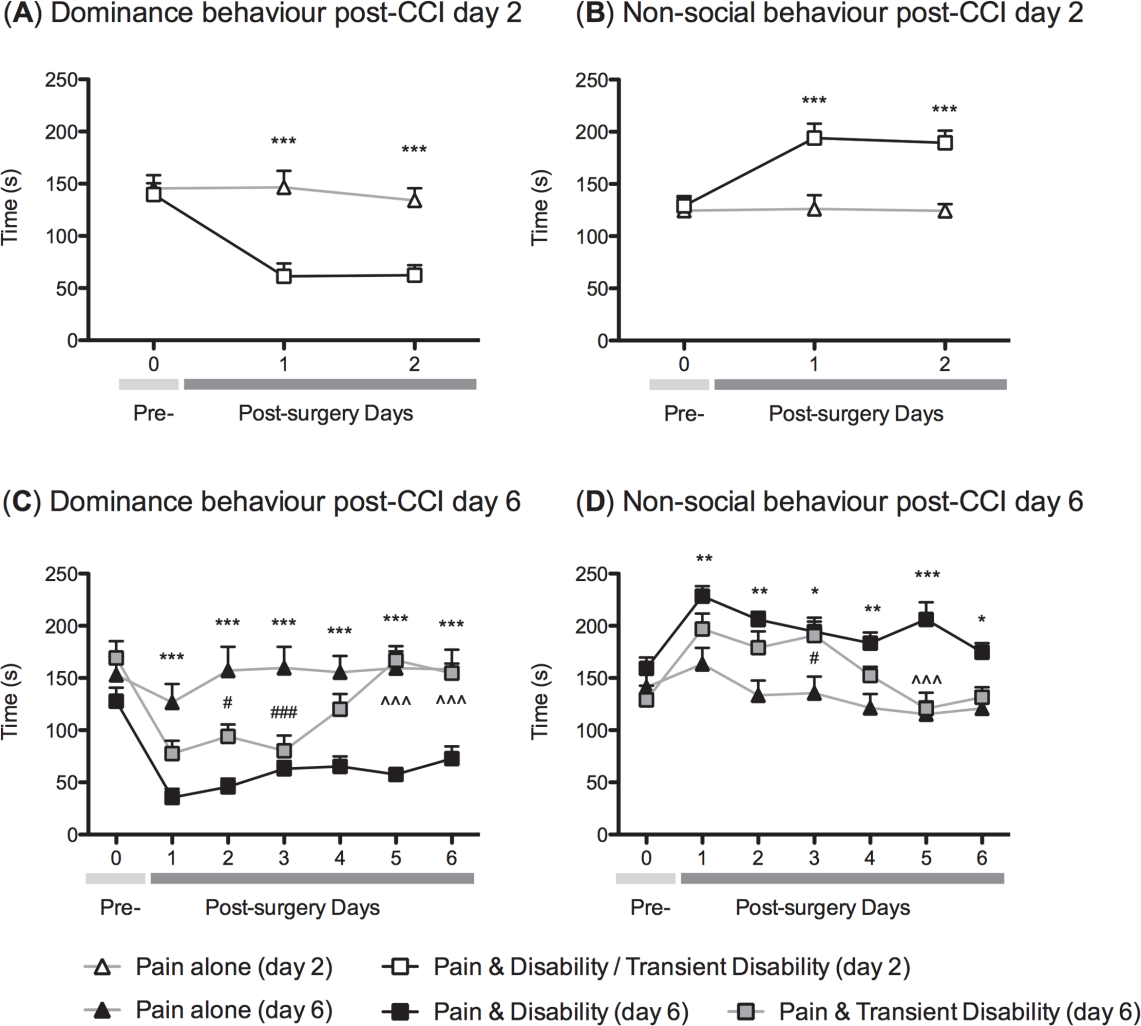
Resident-intruder social interactions testing following chronic constriction injury (CCI) of the sciatic nerve in disability categorised rats. (A&B) Line graphs showing mean (± s.e.m.) duration of dominance and non-social behaviours up to 2 days after CCI, for *Pain & Disability* (n = 9) and *Pain alone* (n = 9) rats. (C&D) Line graphs showing mean (± s.e.m.) duration of dominance and non-social behaviours up to 6 days after CCI, for *Pain & Disability* (n = 9), *Pain & Transient Disability* (n = 9) and *Pain alone* (n = 9) rats. These graphs show pooled behavioural data for all animals used in gene expression studies. Using a two-way ANOVA with a Bonferroni’s post-hoc test; * (P<0.05), ** (P<0.01) and *** (P<0.001) indicate a significant difference between *Pain & Disability* and *Pain alone* rats, ^#^ (P<0.05) and ^###^ (P<0.001) indicate a significant difference between *Pain & Transient Disability* and *Pain alone* rats, ^^^ (P<0.001) indicates a significant difference between *Pain & Disability* and *Pain & Transient Disability* rats.

Two days following nerve injury, in twelve of twenty-seven rats tested (~44%) dominance behaviour was stable on days 1 and 2, these rats were classified as *Pain alone (day 2)*. A further eleven rats (~41%) showed significant reductions (of at least 30%) in duration of dominance behaviours on both post-CCI days, these rats were classified as *Pain & Disability* / *Transient Disability (day 2)*. Four rats could not be classified in into either group. **Fig 1A and 1B** summarises these behavioural outcomes for the 18 resident rats used in subsequent microarray and RT-PCR analyses at day 2. There was a significant reduction in dominance behaviour in the *Pain & Disability / Transient Disability (day 2)* rats compared to *Pain alone (day 2)* rats on both days after CCI (P<0.001), which was mostly accounted for by a significant increase in non-social behaviour (P<0.001).

#### Post CCI Day 6

Six days after CCI, three distinct patterns of behaviour emerged. Firstly, 54 of the 88 (~61%) residents were classified in the *Pain alone (day 6)* group, maintaining dominance behaviours towards the intruder after CCI. Twenty (~23%) of the residents were classified in the *Pain & Disability (day 6)* group, that is, they displayed a significant reduction (at least 30%) in the duration of dominance behaviours on all of the post-CCI days. The remaining 14 residents (~16%) were classified in the *Pain & Transient Disability (day 6)* group, displaying a transient (days 1–3 post-CCI) and significant reduction (~30%) in the duration of dominance, after which behaviour returned to pre-injury levels on days 4–6 after CCI. **Fig 1C and 1D** show the behavioural outcomes, until 6 days after CCI, for the 27 residents used for subsequent gene expression assays. There was a significant reduction in dominance behaviour in the *Pain & Disability (day 6)* rats compared to *Pain alone (day 6)* rats on all 6 post-CCI days (P<0.001), which was mostly accounted for by a significant increase in non-social behaviour (P<0.05-P<0.001). The *Pain & Transient Disability (day 6)* group had a significant reduction in dominance behaviour, and an increase in non-social behaviour on days 1–3 after CCI compared to *Pain alone (day 6)*, but their behaviour returned to normal by days 5 and 6 after CCI.

### Microarray data

Changes in gene expression in L4-L6 spinal cord segments of nerve-injured, behaviourally categorised rats were detected using microarrays at two time points. Two days after CCI the number of probes sets present on the microarrays were 479 in uninjured controls; 486 in *Pain alone (day 2)*; and 477 in *Pain & Disability / Transient Disability (day 2) groups*. Whilst for 6 days after CCI the number of probe sets was, 582 for uninjured controls; 514 in *Pain alone (day 6)*, 586 in *Pain & Disability (day 6)* and 558 in *Pain & Transient Disability (day 6)*. The number of differentially regulated probe sets, did not differ greatly between uninjured controls and the behaviourally categorised rats at either time-point. Variation in the number of genes differentially regulated between replicates at day 2 and at day 6 post-CCI was small and within levels (<2%) considered acceptable using this technology [62].

### Gene expression in the lumbar spinal cord unique to day 2

Two days after CCI a total of 36 genes were identified to have a change in their expression greater than 1.3-fold, with 17 genes up-regulated and 18 genes down-regulated and one regulated both up and down depending on the behavioural grouping, compared to uninjured control lumbar spinal cord (see **Table 2**). Of these 36 genes, 15 were common to both behavioural groups (*Pain alone (day 2)* and *Pain & Disability/Transient Disability (day 2)*), which we have termed ‘injury-dependent genes’. Eight of the genes were up-regulated and 7 down-regulated (**Table 2**). Whilst, 21 genes were identified to be ‘disability-specific genes’, that is they were specifically regulated greater than 1.3-fold in one of the behavioural groups. Eleven of these were selectively regulated in *Pain & Disability/Transient Disability (day 2)* rats (3 up- and 8 down-regulated, **Table 2**), whilst altered regulation of 11 genes was restricted to *Pain alone* rats (7 up- and 4 down-regulated, of which phosphoinositide 3-kinase p85 (PIK3R2) was also up-regulated in *Pain & Disability/Transient Disability (day 2)*).

**Table 2:** A summary of all of the genes up- or down-regulated in the lumbar spinal cord of rats 2 days after CCI determined by MAS v5.0. ALL indicates ‘injury-related’ genes, with regulation occuring in all rats following CCI.

Gene (Symbol)	Direction	Animal Group
^§^Benzodiazepine peripheral receptor (BZRP)	UP	ALL
^**#**^Eukaryotic translation elongation factor 2 (EEF2)	UP	ALL
^§^Glial fibrillary acidic protein (GFAP)	UP	ALL
Glutamine synthetase (GLUL)	UP	ALL
Mitogen activated protein kinase kinase 2 (MKK2)	UP	ALL
^§^Metallothionein 2A (MT2A)	UP	ALL
^**#**^Myelin protein zero (MPZ)	UP	ALL
SNAP-25A	UP	ALL
Agrin (AGRIN)	DOWN	ALL
^§^Catecholamine-O-methyltransferase (COMT)	DOWN	ALL
Cyclin L (CCLN1)	DOWN	ALL
Ectonucleotide pyrophosphatase/phosphodiesterase 2 (ENPP2)	DOWN	ALL
^**#**^Myelin-associated glycoprotein (MAG)	DOWN	ALL
Opioid-like receptor 1 (OPRL1)	DOWN	ALL
^§^Potassium voltage gated channel, Shal-related, member 2 (KCND2)	DOWN	ALL
^‡^Complement component 3 (C3)	UP	P&D+TD
Solute carrier family 18, member 3 (SLC18A3)	UP	P&D+TD
^‡^Phosphoinositide 3-kinase p85 (PIK3R2)	UP / DOWN	P&D+TD / PA
Calcitonin-related polypeptide, beta (CALCB)	DOWN	P&D+TD
Calcium/calmodulin-dependent protein kinase I (CAMK1)	DOWN	P&D+TD
Glutamate receptor, AMPA, alpha 2 (GRIA2)	DOWN	P&D+TD
Neuropeptide Y (NPY)	DOWN	P&D+TD
^**†**^Prostaglandin F receptor (PTGRF)	DOWN	P&D+TD
Tachykinin (TAC1)	DOWN	P&D+TD
Somatostatin (SST)	DOWN	P&D+TD
α-Synuclein (SNCA)	DOWN	P&D+TD
Glutathione-S-transferase, alpha (GST)	UP	PA
^‡^Metallothionein 1A (MT1A)	UP	PA
Monoamine oxidase B (MAOB)	UP	PA
Nestin (NES)	UP	PA
^‡^Sodium channel, type VI, alpha polypeptide (SCN6A)	UP	PA
^‡^Vimentin	UP	PA
^‡^Voltage-dependent anion channel 1 (VDAC1)	UP	PA
Glycine receptor, alpha 1 (GLRA1)	DOWN	PA
^‡^Potassium channel, Shaw-related subfamily, member 1 (KCNC1)	DOWN	PA
^**†**^SRY-box containing gene 10 (SOX10)	DOWN	PA

Whilst, ‘disability-related’ genes, are denoted either, P&D+TD, if they are unique to *Pain and Disability/Transient Disability* rats, or PA, if they are unique to *Pain alone* rats (i.e. non-disabled rats). Symbols denote genes regulated at both 2 and 6 days, specifically:

^§^ persistent regulation in all CCI rats;

^†^ persistent regulation in unique to *Disability*;

^‡^ delayed regulation with respect to *Disability*;

^#^ failure of counter regulation to occur in rats with *Pain and Disability*.

### Gene Expression in the Lumbar Spinal Cord Unique to Day 6

Six days after CCI a total of 61 ‘injury-dependent genes’ were identified to have a significant change in their expression of greater than 1.3-fold compared to uninjured control lumbar spinal cord (see **Table 3**). Forty-two genes were up-regulated and 21 genes down-regulated (including 2 genes that were both up- and down-regulated but in different behavioural groups). Of these 61 genes, 21 ‘injury-dependent genes’ were identified to be specifically regulated in rats from all three behavioural groups compared to control animals, 13 being up-regulated and 8 being down-regulated (see **Table 3**).

**Table 3:** A summary of all of the genes up- or down-regulated in the lumbar spinal cord of rats 6 days after CCI as determined by MAS v5.0.

Gene (Symbol)	Direction	Animal Group
^§^Benzodiazepine peripheral receptor (BZRP)	UP	ALL
Calpain, small subunit 1 (CAPNS1)	UP	ALL
Chemokine (C-C motif) ligand 2 (CCL2)	UP	ALL
^‡^Complement component 3 (C3)	UP	ALL
Dopamine receptor 3 (DRD3)	UP	ALL
^§^Metallothionein 2A (MT2A)	UP	ALL
Gamma-aminobutyric acid B receptor, 1 (GABBR1)	UP	ALL
^§^Glial fibrillary acidic protein (GFAP)	UP	ALL
Glyceraldehyde-3-phosphate dehydrogenase (GAPDH)	UP	ALL
Interleukin 18 (IL-18)	UP	ALL
Interleukin 6 receptor (IL-6R)	UP	ALL
Macrophage migration inhibitory factor (MIF)	UP	ALL
Myelin basic protein (MBP)	UP	ALL
^§^Catecholamine-O-methyltransferase (COMT)	DOWN	ALL
Glutamate receptor, ionotropic, kainate 4 (GRIK4)	DOWN	ALL
Glutamate receptor, N-methyl D-aspartate 2C (GRIN2C)	DOWN	ALL
Heat shock 27kD protein 1 (HSPB1)	DOWN	ALL
^‡^Phosphoinositide 3-kinase p85 (PIK3R2)	DOWN	ALL
^§^Potassium voltage gated channel, Shal-related family, member 2 (KCND2)	DOWN	ALL
*Rattus norvegicus* mRNA for tubulin (TUBG1)	DOWN	ALL
Solute carrier family 24, member 2 (SLC24A2)	DOWN	ALL
Apurinic/apyrimidinic endonuclease 1 (APE1)	UP	P&D
Calmodulin III (CAMIII)	UP	P&D
Calpain 1 (CAPN1)	UP	P&D
Cannabinoid receptor 1 (CNR1)	UP	P&D
Ciliary neurotropic factor (CNTF)	UP	P&D
G protein, beta polypeptide 2-like 1 (GNBL1)	UP	P&D
Galanin (GAL)	UP	P&D
Heat shock 10 kD protein 1 (HSP10)	UP	P&D
Heme oxygenase (HO)	UP	P&D
^‡^Metallothionein 1A (MT1A)	UP	P&D
Monoamine oxidase A (MAOA)	UP	P&D
Neuronal activity-regulated pentraxin (NARP)	UP	P&D
P2X, ligand-gated ion channel, 2 (P2RX2)	UP	P&D
^†^Prostaglandin F receptor (PTGRF)	UP	P&D
Proteasome subunit, alpha type 3 (PSMA3)	UP	P&D
RAB28, member RAS oncogene family (RAB28)	UP	P&D
Ras-related rab1B protein (RAB1B)	UP	P&D
Solute carrier family 1, member 2 (SLC1A2)	UP	P&D
Superoxide dismutase 1, soluble (SOD1)	UP	P&D
Superoxide dismutase 2, mitochondrial (SOD2)	UP	P&D
SWI/SNF related, matrix associated, actin dependent regulator of chromatin, subfamily b, member 1 (SMARCB1)	UP	P&D
^‡^Vimentin (VIM)	UP	P&D
^‡^Voltage-dependent anion channel 1 (VDAC1)	UP	P&D
^#^Myelin protein zero (MPZ)	UP / DOWN	P&D / PA+TD
^‡^Sodium channel, type VI, alpha polypeptide (SCN6A)	UP / DOWN	P&D / PA+TD
ATPase, Ca++ transporting, ubiquitous (ATP2A3)	DOWN	P&D
Glutamate receptor, N-methyl D-aspartate 1 (GRIN1)	DOWN	P&D
Glutamate receptor, N-methyl D-aspartate 2D (GRIN2D)	DOWN	P&D
Glycogen synthase kinase 3 beta (GSK3B)	DOWN	P&D
Neural cell adhesion molecule L1 (L1CAM)	DOWN	P&D
p38 mitogen activated protein kinase (MAPK14)	DOWN	P&D
^‡^Potassium channel, Shaw-related subfamily, member 1 (KCNC1)	DOWN	P&D
Early growth response 1 (EGR1)	UP	PA+TD
Forkhead box M1 (FOXM1)	UP	PA+TD
Mitogen-activated protein kinase kinase 1 (MAP2K1)	UP	PA+TD
^#^Myelin-associated glycoprotein (MAG)	UP	PA+TD
ATPase, Na+K+ transporting, alpha 1 polypeptide (ATP1A1)	DOWN	PA+TD
ATP-binding cassette, sub-family B (MDR/TAP), member 6 (TAP1)	DOWN	PA+TD
^#^Eukaryotic translation elongation factor 2 (EEF2)	DOWN	PA+TD
^†^SRY-box containing gene 10 (SOX10)	DOWN	PA+TD

ALL indicates ‘injury-related’ genes, with regulation occuring in all rats following CCI. Whilst, ‘disability-related’ genes, are denoted either, P&D, if they are unique to *Pain and Disability* rats, or PA+TD, if they are unique to *Pain alone* and *Pain and Transient Disaility* rats (i.e. non-disabled cohort). Symbols denote genes regulated at both 2 and 6 days, specifically:

^§^ persistent regulation in all CCI rats;

^†^ persistent regulation in unique to *Disability*;

^‡^ delayed regulation with respect to *Disability*;

^#^ failure of counter regulation to occur in rats with *Pain and Disability*.

Six days after CCI it is possible to identify a sub-population of rats that have ongoing behavioural disabilities (i.e. *Pain & Disability (day 6)* group), mimicking the presentation of human neuropathic pain patients. Therefore the gene expression patterns unique to this group may be particularly important to the pathophysiology of clinical neuropathic pain. Indeed, 32 ‘disability-specific genes’, were identified as being selectively regulated in only *Pain & Disability (day 6)* rats compared to uninjured controls. Twenty-five of these were up-regulated and 7 down-regulated (**Table 3**). A further 10 genes were selectively regulated in rats without persistent disability (i.e. *Pain alone (day 6)* and *Pain & Transient Disability (day 6)*) 6 days after CCI. Four genes were up-regulated and 6 genes were down-regulated, of which 2 were also up-regulated in *Pain & Disability (day 6)* rats (**Table 3**). Therefore, a total of 40 ‘disability-specific’ genes were identified in the lumbar spinal cord 6 days after CCI.

### Genes regulated at both day 2 and day 6

Of the 80 genes regulated at either day 2 or day 6 post-injury, seventeen genes were regulated at both days 2 and 6. These genes fell into 4 distinct patterns; (i) persistent regulation common to CCI, (ii) delayed gene regulation with respect to disability, (iii) persistent regulation unique to disability and (iv) failure of counter-regulation to occur in animals with *Pain & Disability* (**Tables 2** and **3**).

Five genes displayed persistent regulation common to CCI, the glial fibrillary acidic protein (GFAP, up); metallothionein 2A (MT2A, up); peripheral benzodiazepine receptor (BZRP, up); catecholamine-O-methyltransferase (COMT, down) and potassium channel shal-related member 2 (KCND2, down) genes, were regulated in the same direction on both days common to the CCI, that is they were regulated similarily in all animals at both day 2 and day 6 after CCI.

There were 7 genes which had delayed regulation with respect to the expression of disability following CCI. In the lumbar spinal cord of rats with *Pain & Disability (day 6)* the genes for vimentin (VIM), voltage dependent anion channel 1 (VDAC1), metallothionein-1A (MT1A), and sodium channel type VI alpha (SCN6A), had up-regulation delayed until day 6 after CCI, contrastingly animals with *Pain alone (day 2)* display a similar upregulation earlier at day 2. As a result of the delayed up-regulation of SCN6A in *Pain & Disability (day 6)* rats, until day 6 after CCI, they differ from *Pain alone (day 6)* and *Pain & Transient Disability (day 6)* rats, which by day 6 displayed a subsequent down-regulation. Conversely, potassium channel shaw-related member 1 (KCNC1), had delayed down-regulation in the lumbar spinal cord of animals with *Pain & Disability (day 6)* until day 6 after CCI. There was delayed up-regulation of complement component 3 (C3) in rats with *Pain alone* and a delayed down-regulation of phosphoinositide 3-kinase p85 (PIK3R2) in *Pain & Disability* rats (which actually increased), when changes in day 2 post-CCI rats were compared to rats at day 6 post-CCI.

Two changes in gene expression were found to occur exclusively in a single behavioural group. SRY-box containing gene 10 (SOX10) was down-regulated at both day 2 and day 6 after CCI in the spinal cord of rats without disability (*Pain alone* and *Pain & Transient Disability* groups). Whilst the gene for prostaglandin F receptor (PTGRF) was selectively regulated in the spinal cord of rats with *Pain & Disability*, initially being down-regulated 2 days after CCI, but subsquently up-regulated by day 6.

Three genes, eukaryotic translation elongation factor 2 (EEF2, up), myelin protein zero (MPZ, up) and myelin-associated glycoprotein (MAG, down), were regulated similarily in the lumbar spinal cord of all rats 2 days after CCI, but subsequently at day 6 the direction of regulation selectively changed in only rats without disability (*Pain alone* and *Pain & Transient Disability* groups). Thus, these genes can be considered ‘injury-dependent’ at day 2 and ‘disability-specific’ at day 6.

The remaining 63 genes were regulated selectively in the lumbar spinal cord at only day 2 or day 6 after CCI (see **Tables 2** and **3**). Nineteen of these changes in gene regulation were found to occur at only day 2 following sciatic CCI, with 7 ‘injury-dependent’ genes and 12 ‘disability-specific’ genes (8 genes regulated selectively in *Pain & Disability / Transient Disability* rats and 4 genes regulated in rats with *Pain alone*). At day 6 following CCI, 44 genes were specifically regulated, 14 ‘injury-dependent’ genes, and 30 ‘disability-specific’ genes (25 genes selectively regulated in *Pain & Disability* rats, and 5 genes regulated in rats with *Pain alone* and *Pain & Transient Disability*).

**Fig 2:**
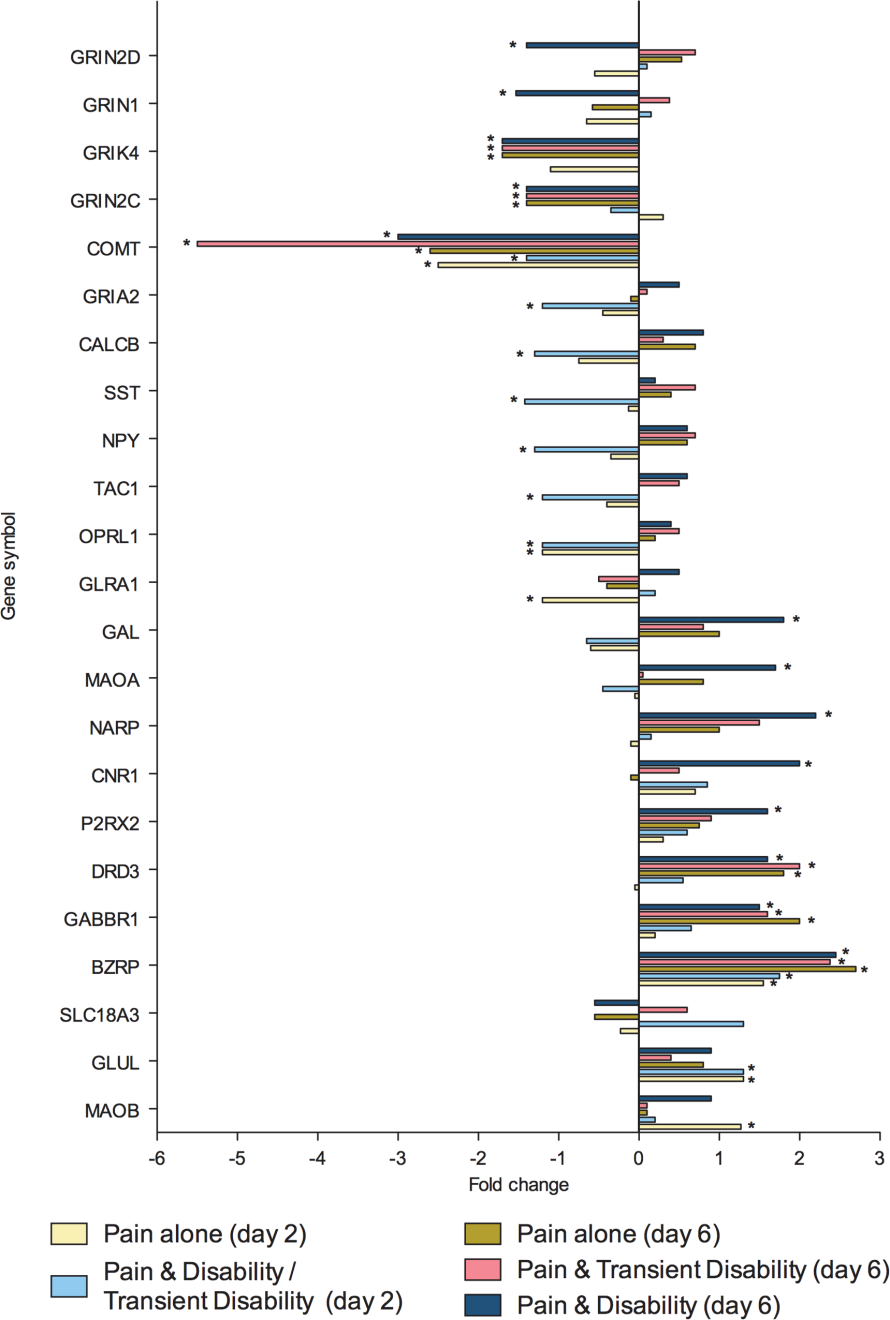
Gene transcripts, which encode for proteins involved in neurotransmission, that are specifically regulated in the lumbar spinal cord after CCI, as determined by microarray analysis. Changes in gene expression have been tested at both 2 and 6 days after CCI on pooled mRNA samples from each disability group, compared to uninjured controls. * Indicates up- or down-regulation determined by MAS v5.0.

**Fig 3:**
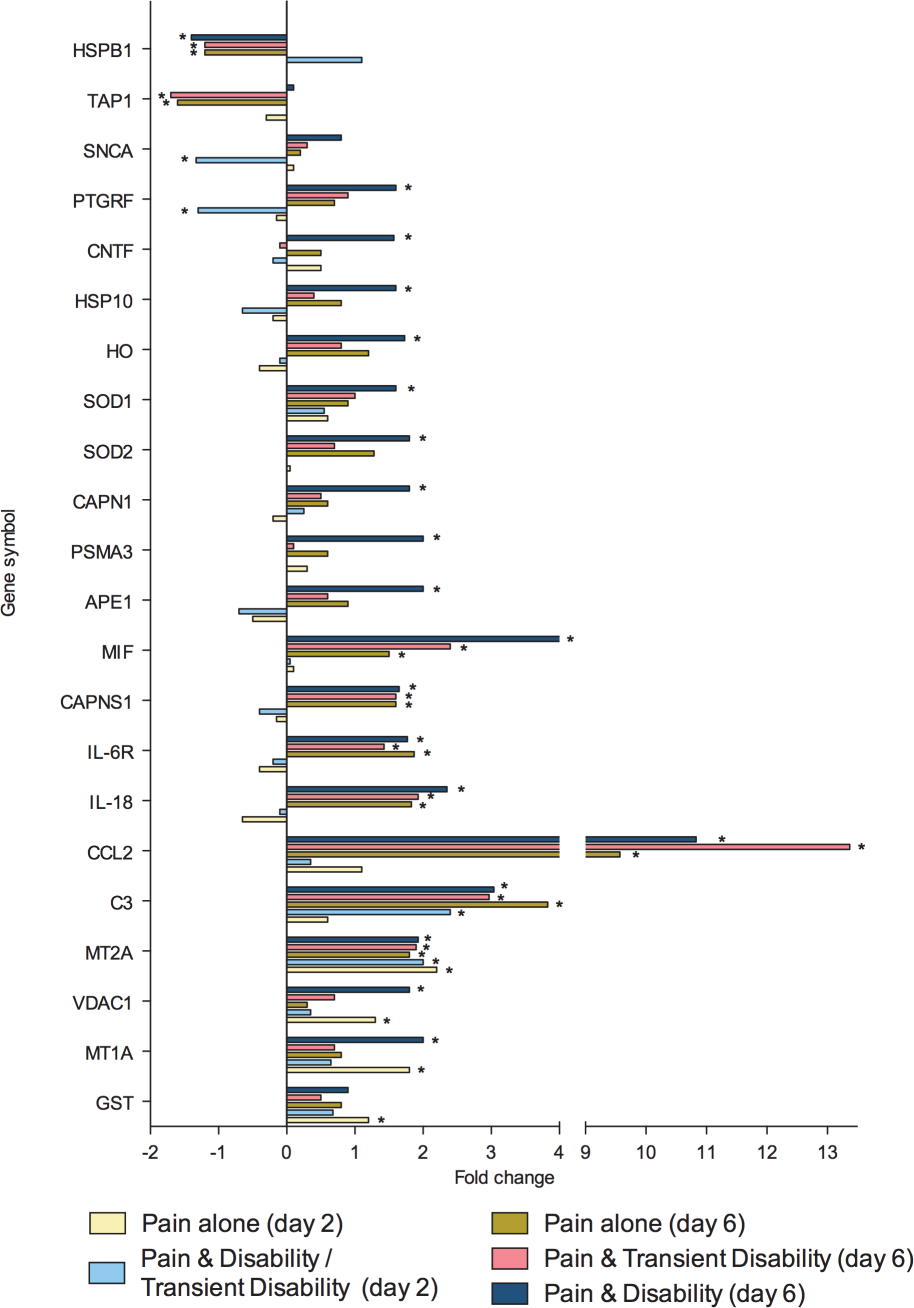
Gene transcripts, which encode for proteins involved in inflammation and/or cellular stress, that are specifically regulated in the lumbar spinal cord after CCI, as determined by microarray analysis. Changes in gene expression have been tested at both 2 and 6 days after CCI on pooled mRNA samples from each disability group, compared to uninjured controls. * Indicates up- or down-regulation determined by MAS v5.0.

**Fig 4:**
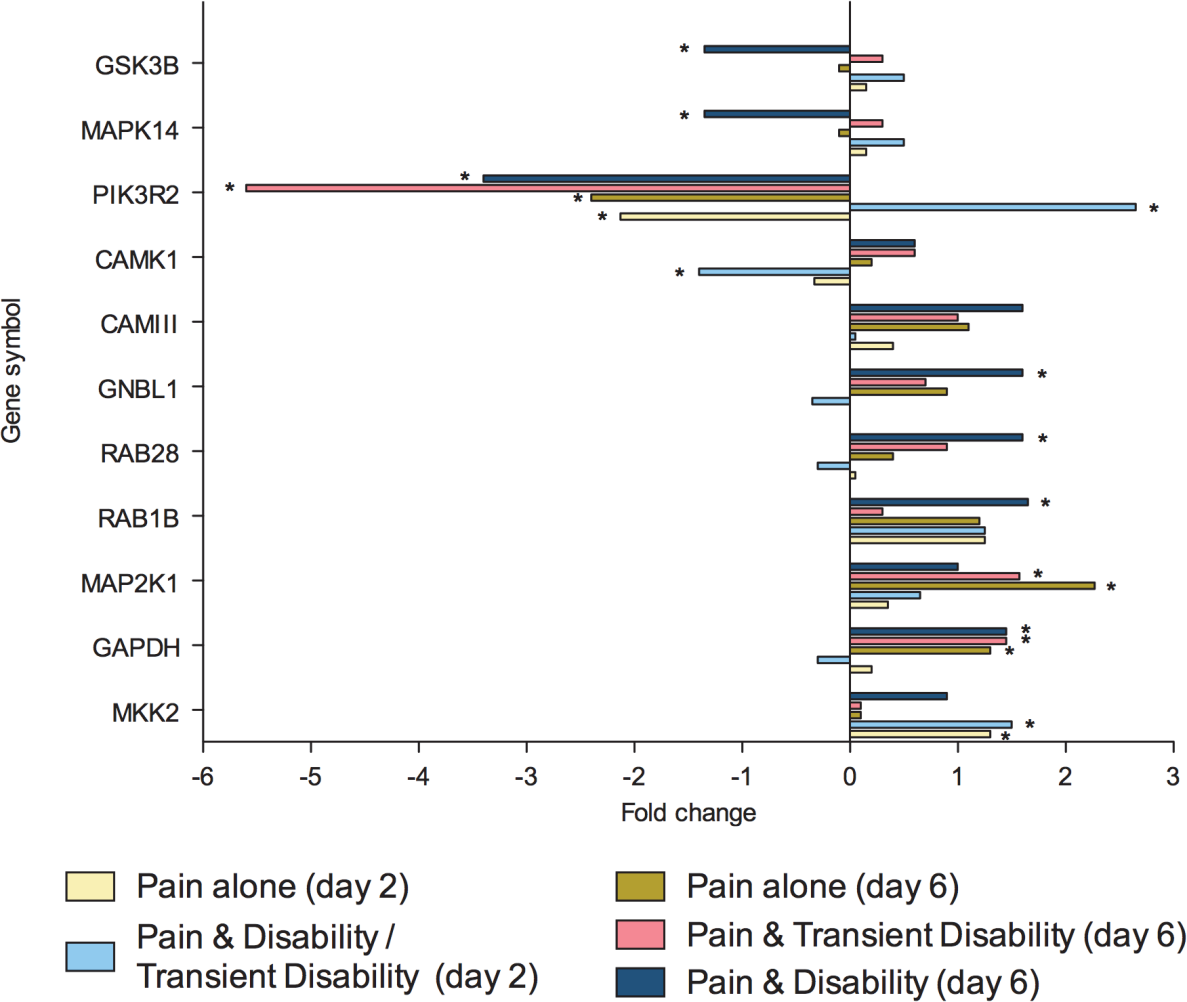
Gene transcripts, which encode for proteins involved in cellular signalling, that are specifically regulated in the lumbar spinal cord after CCI, as determined by microarray analysis. Changes in gene expression have been tested at both 2 and 6 days after CCI on pooled mRNA samples from each disability group. * Indicates up- or down-regulation determined by MAS v5.0.

**Fig 5:**
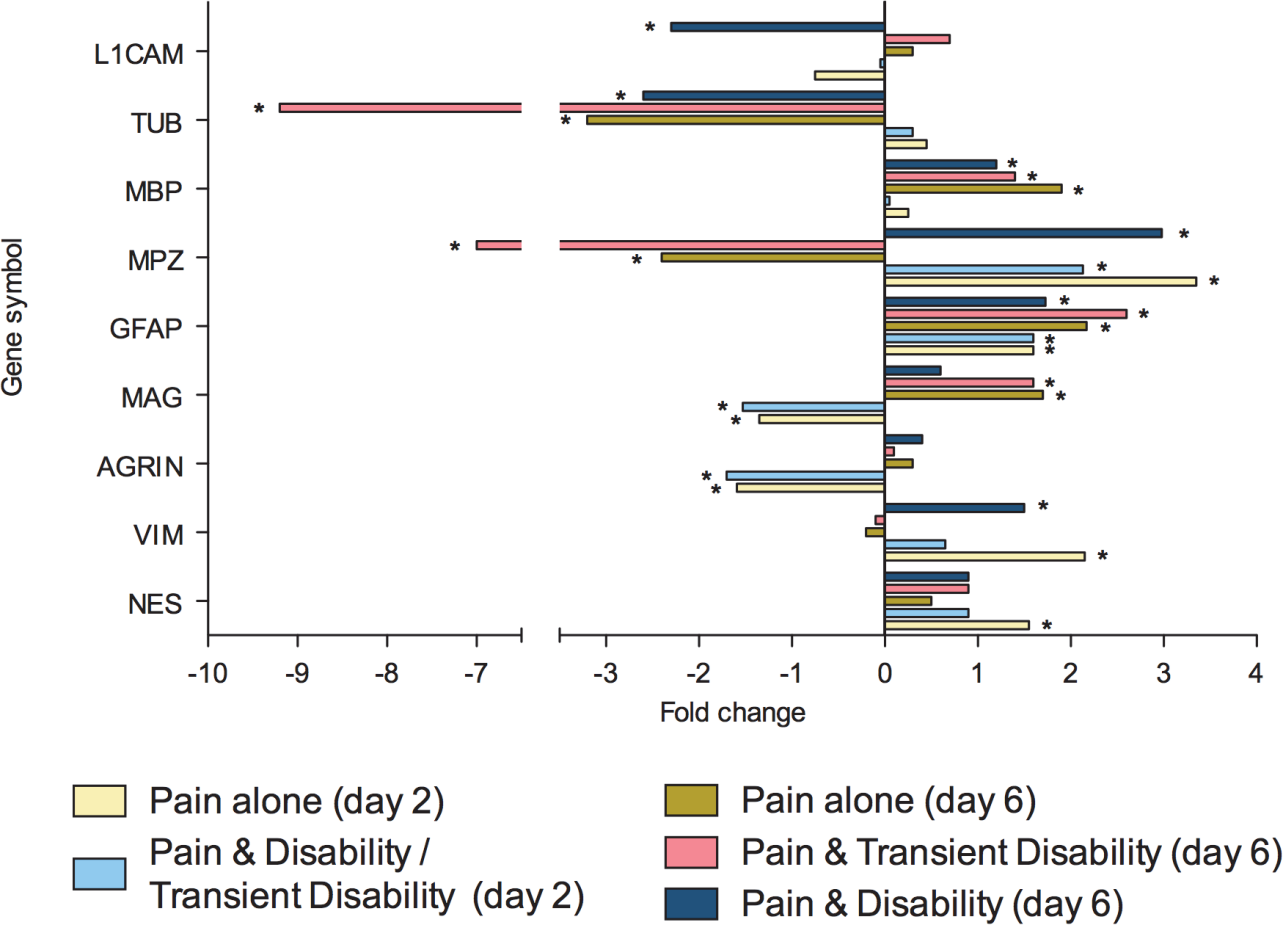
Gene transcripts, which encode for proteins involved in cellular structure, that are specifically regulated in the lumbar spinal cord after CCI, as determined by microarray analysis. Changes in gene expression have been tested at both 2 and 6 days after CCI on pooled mRNA samples from each disability group, compared to uninjured controls. * Indicates up- or down-regulation determined by MAS v5.0.

**Fig 6:**
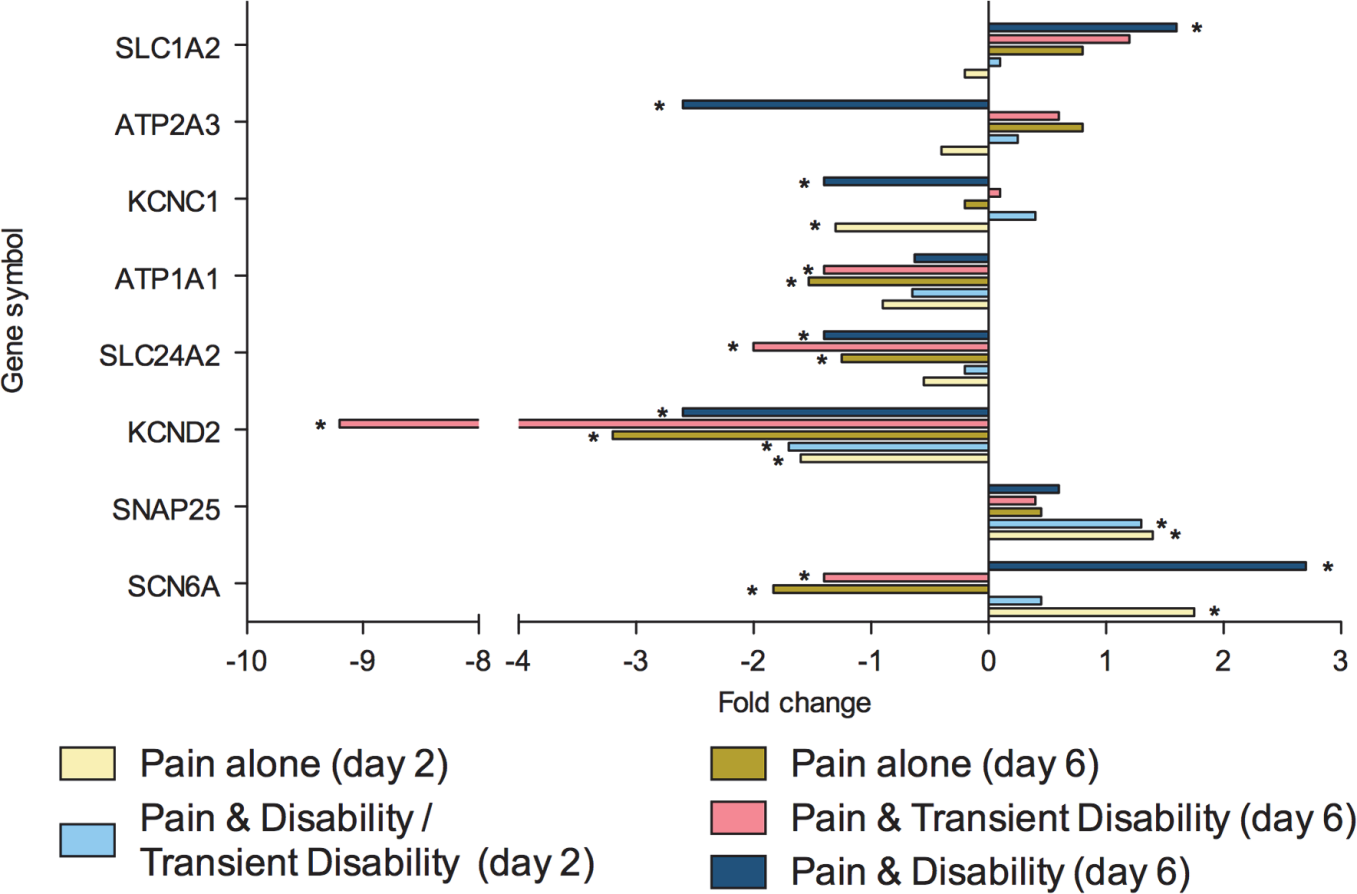
Gene transcripts, which encode for proteins involved in ionic balance, that are specifically regulated in the lumbar spinal cord after CCI, as determined by microarray analysis. Changes in gene expression have been tested at both 2 and 6 days after CCI on pooled mRNA samples from each disability group, compared to uninjured controls. * Indicates up- or down-regulation determined by MAS v5.0.

**Fig 7:**
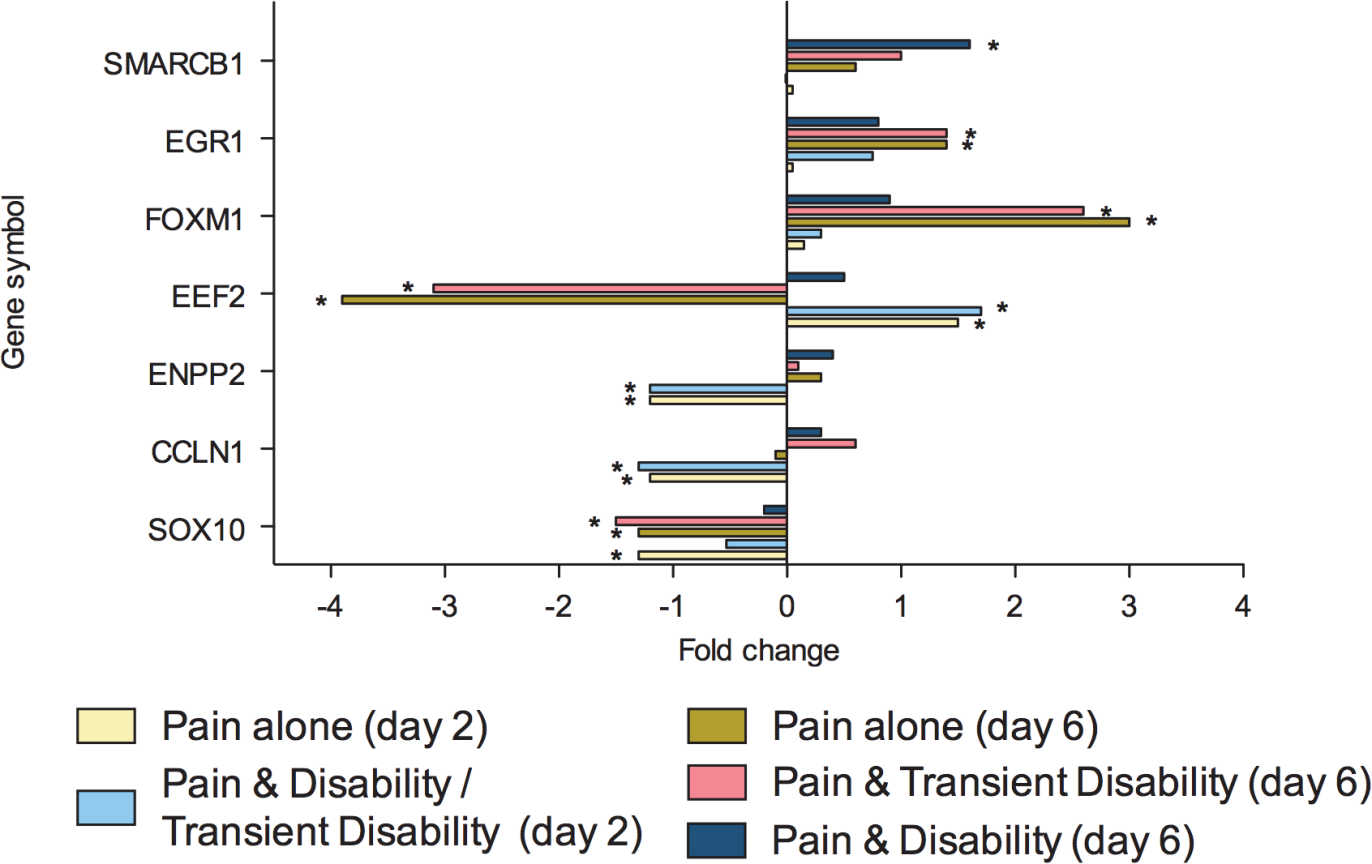
Gene transcripts, which encode for proteins involved in transcriptional or translational processes, that are specifically regulated in the lumbar spinal cord after CCI, as determined by microarray analysis. Changes in gene expression have been tested at both 2 and 6 days after CCI on pooled mRNA samples from each disability group, compared to uninjured controls. * Indicates up- or down-regulation determined by MAS v5.0.

To summarise, at both post injury days 2 and 6, eighty genes were regulated greater than 1.3-fold in the lumbar spinal cord (**Tables 2** and **3**). Of these genes, 26 were strictly ‘injury-dependent’; 49 were strictly ‘disability-specific’; 3 genes were initially ‘injury- dependent’, changing to ‘disability-specific’ by day 6; whilst 2 genes were ‘disability-specific’ at day 2 changing to ‘injury-dependent’ by day 6. All genes were subsequently categorised according to the function of the protein which they encoded; neurotransmission (n = 23, **Fig 2**), inflammation and/or cellular stress (n = 22, **Fig 3**), cellular signalling (n = 11, **Fig 4**), cellular structure (n = 9, **Fig 5**), ionic balance (n = 8, **Fig 6**) and transcriptional and translational processes (n = 7, **Fig 7).** Because our overall aim was to begin to describe the spinal adaptations specific to altered activity in spinal neurons that might drive affective-motivational change, we proceeded to interrogate the transcriptional changes in the neurotransmission functional group using RT-PCR.

### Real-Time RT-PCR Data

RT-PCR was used to confirm the microarray results for 17 genes primarily from the neurotransmission functional group. These genes were chosen based on their significant changes in gene expression, as well as having a functional role in neurotransmission in the nociceptive pathways of the spinal cord. Of the 17 genes tested, 10 (59%) showed changes in expression pattern consistent with the changes identified by microarray analysis. Overall 7 genes were found to be ‘injury-dependent’ being modulated in all nerve injury rats and 4 genes were ‘disability-specific’ being selectively regulated in only *Pain & Disability* animals. Although, perfect congruence was not expected due to the inherent differences in sensitivity and dynamic range between the two techniques, overall, the 59% agreement is in the expected range, based on similar studies [49, 63, 64].

**Fig 8:**
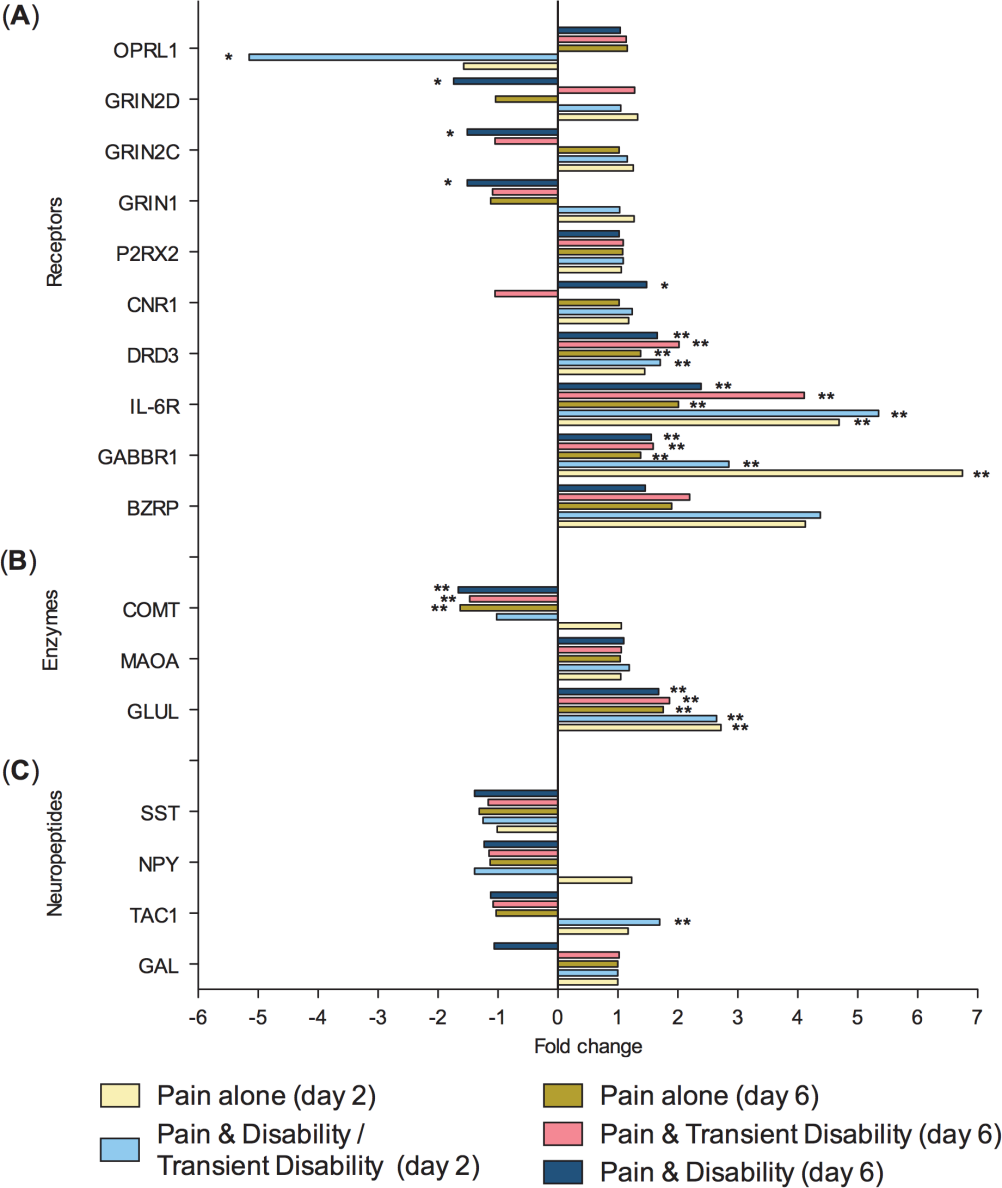
Gene transcripts that are significantly regulated in the lumbar spinal cord after CCI, as determined by real time PCR. Changes in gene expression have been tested at both 2 and 6 days after CCI on pooled mRNA samples from each disability group, compared to uninjured controls. Genes encoding; (A) receptors; (B) neurotransmitter degradation enzymes; and (C) neuropeptides. Statistically significant changes indicated by; * P<0.05 and **P<0.005 (Pairwise Fixed Reallocation Randomisation Contest).

When the genes examined by real time RT-PCR were sub-divided into functional categories, changes in receptor genes showed a high degree of congruence. Changes in 8 out of 10 receptor genes detected by real time RT-PCR (**Fig 8A**), were consistent with the microarray findings. Of these 8 genes, 5 were ‘injury-dependent’ showing altered expression patterns in all nerve injured rats. Two of the ‘injury- dependent’ genes showed identical patterns to the microarray, with BZRP up-regulated at both time points and opioid-like receptor 1 (OPRL1) down-regulated only at day 2. The other three ‘injury-dependent’ genes, the GABA_B_ (GABBR1), interleukin-6 (IL-6R) and dopamine 3 (DRD3) receptors, showed up-regulation at both days 2 and 6, whereas the microarray results indicated an increase only at day 6. Three genes, were confirmed by real time RT-PCR to be ‘disability-specific’ as in the microarray, being selectively regulated in only *Pain & Disability (day 6)* rats at day 6; these were, cannabinoid receptor 1 (CNR1,up), NMDA subunits NR1 (GRIN1, down) and NR2D (GRIN2D, down). NMDA receptor subunit NR2C (GRIN2C) was previously identified as an ‘injury-dependent’ gene in the microarray, however PCR revealed it to be a ‘disability specific’ gene, as it was down-regulated at day 6 in *Pain & Disability (day 6)* rats. Given the RT-PCR analysis included mRNA from additional CCI rats, it likely reflects a more accurate picture of the regulation of this gene. The only gene of the receptor class which no longer showed any significant change in expression in the RT-PCR analysis, was P2RX2.

Consistent with microarray findings, neurotransmitter degradation enzymes, COMT and glutamine synthase (GLUL), were confirmed as ‘injury-dependent’ genes by real time RT-PCR in (**Fig 8B**). With that said, down-regulation of COMT, was only congruent at day 6, with no change in gene expression at day 2 after nerve injury. Whilst the up-regulation of GLUL was identified at both timepoints, in contrast to the up-regulation restricted to day 2 in the microarray analysis. The up-regulation of monoamine oxidase A (MAOA) in the *Pain & Disability* group was not detected by real time RT-PCR.

Four ‘disability-specific’ genes for neuropeptides identified in the microarrays were not confirmed in real time RT-PCR (**Fig 8C**).

## Discussion

We identified using microarrays, a total of 80 genes that were specifically regulated in the lumbar spinal cord in response to CCI. At 2 and 6 days after injury, 26 genes were considered ‘injury-dependent’ showing altered expression in all rats, whilst there were 54 ‘disability-specific’ genes, regulated in one or more of the disability groups. Using real time-PCR, we confirmed the expression pattern of 7 ‘injury-dependent’ genes and 3 ‘disability-specific’ genes from the microarrays, plus discovered the NMDA NR2C gene having ‘disability-specific’ regulation. These findings provide insight into the temporal and dynamic response of gene expression with regards to the development of both sensory abnormalities and disabilities following nerve injury. The significance of our discovery of ‘disability-specific’ genes is emphasized by the fact that forty percent of the genes identified in our study have been previously identified as ‘pain genes’ according to the pain gene database (22 genes) and nineteen of the genes have been identified in a recent and comprehensive ‘pain gene’ meta-analysis [49, 65]. This is the first suggestion however, of a role for these genes in the expression of disability in neuropathic conditions.

### Injury-dependent gene changes

Microarrays were used to detect spinal cord gene regulation common to CCI rats in the immediate (day 2) and early (day 6) stages of injury. CCI triggers a cascade of neural and inflammatory changes along the neuraxis leading to sensory hypersensitivity [25, 27]. It triggers a loss of predominantly myelinated nerve fibres distal to the ligation and Wallerian degeneration, sensitisation to chemical and inflammatory mediators, altered phenotypes of surviving fibres, sprouting of sympathetic fibres and ectopic firing [66]. These changes are initially adaptive, promoting injury healing by signalling acute nociception and through immune-mediated repair and removal of cellular debris. However if these processes persist they can lead to central sensitisation, where neurons in the spinal cord dorsal horn change their phenotype, through altered gene expression, leading to nociceptive signalling in the absence of tissue damage. Many of these mal-adaptive changes underlie the characteristic changes in sensation associated with sensory-discriminative aspects of neuropathic pain, such as allodynia, hyperalgesia, and spontaneous pain, and changes in expression of several ‘pain genes’ have been associated with these. The changes in gene expression identified in all animals following CCI, likely play a role in these sensory-discriminative abnormalities, which are seen in all disability groups after CCI [25, 27]. Of the 26 ‘injury-dependent’ genes identified, at least 18 have a known role in nociception or the development and/or maintenance of sensory abnormalities after nerve injury. Although we cannot rule out that some of the changes in expression of these 26 genes may represent the response to resident-intruder testing, this seems unlikely given that such a large proportion of these genes have already been implicated in sensory abnormalities. Below we discuss these ‘injury-dependent’ genes.

#### Genes identified in both microarray and RT-PCR

Substantial changes in spinal cord genes involved in neurotransmission were hypothesised given their importance in the mechanisms of central sensitisation and its contribution to sensory abnormalities, which characterise pain following nerve damage. The changes identified in this study included, the gene for the GABA_B_ receptor 1 (GABBR1) which was up-regulated, consistent with the findings of McCarson and colleagues [67], although down-regulation has been reported at later time-points [49, 68, 69]. A loss of GABAergic tone is reported in neuropathic pain states [70–72], and activation of these receptors is antinociceptive [73, 74]. Thus, the up-regulation of spinal GABAR1 receptor mRNA reported by us, and others [67] may reflect an initial compensatory response to the loss of spinal cord GABA.

The up-regulation of the dopamine 3 (D_3_) receptor gene (DRD3) at day 6 following CCI may also play a significant role in modulating spinal sensory mechanisms. A growing body of literature supports a role for dopamine, and D_2-like_ receptors (D_2_, D_3_ and D_4_), in the descending inhibition of nociceptive signalling in the superficial layers of dorsal horn. D_2-like_ receptor agonists suppress nociceptive responses [75–78], therefore up-regulation of D_3_ may reflect a compensatory response to injury-evoked over-activity of descending inhibitory pathways.

A transient down-regulation of the expression of the opioid-like 1 receptor (OPRL1) identified at day 2 only, may contribute to the development of sensory abnormalities in the initial period after injury. However expression patterns may well change at later time-points (see [69, 79]). The modulation of nociception by the OPRL1 is supported by observations from N/OFQ-R^-/-^ mice [80]; behavioural neuropharmacological studies [81–84]; and functional anatomical observations [85]; however the precise roles for this receptor in the expression of sensory changes following CCI remain to be systematically explored.

The COMT enzyme is a key modulator of dopaminergic and adrenergic neurotransmission through its metabolic actions on catecholamines. In humans, the val^158^met polymorphic variant has a 3–4 fold lower activity [86], leading to increased pain sensitivity and affective ratings of pain [87, 88], and is associated with several chronic pain conditions [89]. Systemic administration of COMT inhibitors is pro-nociceptive, exacerbating pain in inflammatory pain models via a β2/3-adrenergic mechanism; the dorsal horn of spinal cord, is one of several locations at which this may occur [90, 91]. The superficial dorsal horn of the spinal cord has a high expression of COMT [92], and the decrease in COMT mRNA in all rats after CCI, may thus contribute to the maintenance of the sensory abnormalities, via locally increased catecholamines and through β2/3-adrenergic receptors. In neuropathic conditions systemically administered COMT inhibitors reduce pain hypersensitivity [93, 94]. However, our data suggest that this effect is unlikely to be mediated at the level of the spinal cord.

The glutamate-glutamine shuttle, which allows recycling of extrasynaptic glutamate, and is catalysed by astrocytic glutamine synthetase (GLUL), has been implicated in persistent pain [95, 96]. Moreover, GLUL inhibition has been shown to reduce nocifensive behaviour in a model of inflammatory pain [97]. Our microarray and PCR findings, that GLUL expression, is up-regulated following nerve injury in all rats, is in keeping with previous reports of increased expression in the hypoglossal nucleus following hypoglossal nerve transection [98]. In this study the authors hypothesised that this may be protective against the excitotoxic effects of the excess of glutamate released following injury. Hence, the increase in glutamine synthetase may be in response to excess glutamate release into the dorsal horn after CCI, more generally changes in glutamate and GABA synthesis appears to promote central sensitisation, contributing to sensory abnormalities [95–97].

Our findings that peripheral benzodiazepine receptor (BZRP) expression is up-regulated in all rats, 2 and 6 days after CCI is consistent with previous findings, where expression in the spinal cord peaked at day 3 [46]. Further the sensory abnormalities evoked by nerve injury were reversed by treatment with a BZRP antagonist and were associated with similar increases in BZRP expression. The reversal of the sensory abnormalities, depended on inhibition of production of the steroids, allopregnanolone and 3α,21-dihydroxy-5α-pregnan-20-one (3α,5α-THDOC), positive allosteric modulators and activators of the GABA_A_ receptor [99]. The BZRP enhances the activity of the mitochondrial permeability transition pore (MPTP), and is involved in steroid production, cell survival and inflammatory processes [100, 101]. BZRP is also expressed by microglia, and has been reported to be significantly up-regulated in response to neuroinflammation [102]. BZRP is involved in the induction of apoptosis following exposure to cytokines, such as TNF [100], an environment consistent with the dorsal horn of the spinal cord after nerve injury.

Given that neuropathic pain due to nerve injury is now considered a neuro-immune disorder [103], it is no surprise that many genes involved in inflammation are up-regulated in the dorsal horn of the spinal cord following nerve injury. The mechanism of action of these inflammatory mediators is generally to increase glial cell activation, as well as to increase firing rates of neurons either directly or to sensitise them to other neurotransmitters.

Interleukin-6 (IL-6) is a major pro-inflammatory cytokine, expressed by neurons, microglia and astrocytes [103]. Nerve injury has previously been shown to increase up-regulation of IL-6 at multiple sites along the neuraxis, particularly the dorsal horn of the spinal cord [104, 105]. Evidence for long term up-regulation of the IL-6 receptor mRNA in the spinal cord has also been reported after CCI [106]. Although IL-6 and its receptor play a critical role in nerve regeneration [107, 108], they are also central to the development and maintenance of sensory abnormalities [104, 109–112]. Thus, the increase in IL-6R mRNA reported here, in all rats post-CCI is likely to potentiate nociceptive signalling.

#### Genes identified using the microarrays

A range of inflammatory-related genes were uncovered in the microarrays, but were not subjected to confirmation using real time PCR. The significant congruence of these data with that reported in a number of earlier published reports as well as a recent meta-analysis confirms the likely importance of our findings.

A massive (57-fold) up-regulation of spinal cord expression of the chemokine CCL2 following CCI has previously been reported on day 14 after CCI, as well as in four independent microarray studies [49], confirming the robust comparability of our data with the existing literature. We identified a 9-fold increase in CCL2 mRNA in the dorsal horn of the spinal cord in all CCI rats by day 6, the largest increase in any gene on our microarray. CCL2 has been implicated in sensory abnormalities following nerve injury through recruitment and activation of microglia and direct excitation of spinal cord neurons [113–116].

Increased expression of glial fibrillary acidic protein (GFAP) following nerve injury has been documented in six independent microarray studies in models of neuropathic pain, as well as real time RT-PCR after CCI [49], the significant up-regulation seen in all our nerve injured rats fits well with these data. GFAP is a well-characterised structural protein associated with astrocyte activation. In general central activation of astrocytes is associated with development and maintenance of sensory abnormalities, through the production and release of inflammatory mediators [103]. Furthermore, the increases in the pro-inflammatory cytokine, IL-18, and macrophage migration inhibitory factor (MIF) reported here have also previously been shown to increase their spinal cord expression following nerve injury, and have been implicated in contributing to sensory abnormalities [117–120].

These examples of comparable findings across studies adds confidence to the significance and importance of the disability-specific genes defined in the microarray and RT-PCR analyses, which are now considered.

### Disability-specific gene changes

Changes in affective-motivational state are an integral aspect of the pain experience, manifesting in behavioural disturbances described as disabilities in many neuropathic pain sufferers. Changes in gene expression at multiple levels of the neuraxis, including the first synapse are likely to underpin the onset of such problems. In rats, sciatic nerve CCI triggers distinct patterns of behavioural change, which may be driven in part by altered regulation of gene expression in L4-L6 spinal cord structures (neurons, glial cells, vasculature), which selectively influences spinal neurons with supra-spinal targets. Overall, we have identified 54 ‘disability-specific’ genes, which are either uniquely regulated in rats with *Pain & Disability*, or in rats without persistent disability (*Pain alone* / *Pain & Transient Disability*), compared to uninjured controls. Each gene showed a unique temporal and dynamic pattern of regulation in the spinal cords of rats from each disability sub-population. These differential patterns of gene expression may be central to the development of complex behavioural changes, such as reduced dominance in social interactions, disrupted sleep-wake cycles, and disrupted HPA and HPT axes, seen in the *Pain & Disability* cohort [26, 27, 31, 32].

Whilst twenty-one of the 54 ‘disability-specific’ genes have previously been suggested to contribute to acute nociception or the sensory abnormalities following nerve-injury, a role for these genes in the affective-motivational aspects of pain is a novel possibility suggested by our data. Indeed, to our knowledge no studies outside our laboratory have attempted to compare gene expression changes in sub-populations of rats displaying markedly different behavioural responses following nerve injury. Below we discuss these ‘disability-specific’ genes in the context of the function of their protein product.

#### Genes identified in both microarray and RT-PCR

Neurotransmitter genes: Several genes involved in glutamate neurotransmission were uniquely regulated in rats with disrupted social interactions (i.e., disability). The AMPA receptor 2 subunit (GRIA2), is reported to be downregulated in the L4-L6 spinal cord 24 hours after hindpaw inflammation and after 2 days in the ventral horn following sciatic nerve crush [121, 122]. After CCI there are increased protein levels for AMPA receptor subunits in the dorsal horn at 4 and 8 days after CCI [123]. Our data showed a select reduction of GRIA2, mRNA expression 2 days after injury in rats with *Pain & Disability*, which returns by day 6.

The NMDA receptor consists of heterotetrameric assemblies comprised most commonly of two NR1 (GRIN1) subunits, and two NR2A-D (GRIN2A-D,) subunits. Following CCI, NR1 and NR2 mRNA expression has been reported to increase in the dorsal horn ipsilateral to the injury from day 3 post-injury up to day 14 [124]. Although the specific NR2 subunit was not specified, interrogation of the primer sequences indicated it was the GRIN2A gene (NR2A) that was measured. Twenty-one days after spinal nerve ligation (SNL) dorsal horn expression of the NR2D subunit (mRNA and protein) was no different to that of uninjured rats [125]. In the ventral horn, sciatic nerve transection leads to a decrease in NR1, NR2B and NR2D mRNAs and NR1 protein [126], highlighting a possible role for axotomy in triggering these ventral horn changes in gene regulation. We are not aware of any studies to date that have investigated the regulation of the NR2C mRNA in response to peripheral nerve injury. Our data showed decreased NR1 (GRIN1), NR2C (GRIN2C) and NR2D (GRIN2D) mRNA in the L4-L6 spinal cord of rats with *Pain & Disability* six days after CCI. Whether these changes in mRNA are translated into protein/s is the next step in determining the functional significance of these gene expression data. For example, it would be critical to define how a reduction in expression of NR1 and NR2C/D subunits in *Pain & Disability* rats might affect glutamatergic receptor transmission in L4-L6 spinal segments. NMDA receptors with NR2A/B subunits are more sensitive to Mg^2+^ block and have a more rapid EPSC decay, than receptors containing NR2C/D subunits [127, 128]. Thus, NR2A/B containing receptors deactivate faster than those with NR2C/D subunits. The expression of NR2C/D subunits is restricted to inhibitory interneurons in the superficial dorsal horn, unlike NR2A/B which appear ubiquitous [129]. It is clear that several AMPA and NMDA receptor subunit genes have ‘disability-specific’ spinal cord expression after CCI. These receptor expression patterns likely cause alterations in glutamatergic signalling, which ultimately promotes activity in ascending pathways, which drive the expression of behavioural disabilities.

The cannabinoid receptor 1 (CNR1) is highly expressed by both neurons and glia in superficial dorsal horn of the spinal cord [130]. In experimental groups using small numbers of rats, protein levels for the CNR1 receptor in the lumbar dorsal horn are reported to increase after CCI [131, 132]. Further, CNR1 mRNA has been reported to increase in other models of neuropathic pain in several microarray studies [49, 131]. Here we confirm upregulation of spinal CNR1 mRNA, but only in the spinal cord of *Pain & Disability* rats. Therefore CNR1 may have an as yet undetermined role in the expression of disability. The increase in CNR1 mRNA in a select subgroup of rats was unexpected since it is suggested to enhance anti-nociception, however we have previously reported that allodynia is equal across all disability rats [25, 27]. Furthermore, an anti-nociceptive role is well established, with endogenous cannabinoids, as well as exogenously applied cannabinoid agonists, consistently reported to attenuate hyperalgesia and allodynia following nerve injury [131, 133–135].

Monoamine oxidase isoforms A (MAOA) and B (MAOB) metabolise noradrenaline and dopamine as well as serotonin (5-HT) and are found in both neurons and glia. Lumbar spinal MAOA levels increase following CCI, which can result in decreased spinal 5-HT concentrations [136]. Decreased binding at spinal 5HT_3_ receptors has been suggested to both enhance [137] and decrease [136, 138] nociception. However, monoamine oxidase inhibitors are antinociceptive in models of neuropathic pain [136, 139] and analgesic in humans where they have been prescribed for comorbid depression [140, 141]. Our microarrays revealed that at day 6 post-CCI, MAOA mRNA was increased in rats with *Pain & Disability* and MAOB mRNA was increased in rats that did not develop disabilities at 2 days after injury (i.e., *Pain alone*). This is the first report of complementary changes in spinal MAOA and MAOB mRNA expression in behaviourally distinct subgroups of rats after CCI [136]. This temporally distinct and ‘disability-specific’ pattern of MAO isoform expression may play a role in differentially shaping the activity of ascending supraspinal pathways known to be involved in the affective-motivational component of pain.

#### Genes identified using the microarrays

Inflammatory mediator genes: In addition to genes identified in the neurotransmission group, large numbers of disability-specific genes regulated from the inflammation functional group were identified. Thus, the expression of disability may be driven in part by a specific immune response. This suggestion is supported by the agreement of findings from earlier studies and the present findings. For example, complement component 3 (C3) is part of the innate immune system. C3 mRNA is upregulated after CCI, and C3 contributes to development of sensory abnormalities [46, 49, 142]. We confirmed C3 mRNA upregulation in all rats by day 6, however there is an earlier up-regulation in *Pain & Disability* rats, suggesting a more rapid complement activation in this subgroup.

Prostaglandin F (PTGRF) receptors are known to be involved in the development of ATP-induced allodynia, and are co-localised with P2X_2/3_ (P2RX2) receptors in spinal cord neurons [143]. Our data showed the PTGRF receptor gene was initially down-regulated at day 2 in *Pain & Disability* rats, before both the PTGRF and P2RX2 genes were up-regulated again in only *Pain & Disability* rats at day 6. P2X receptors play a role in allodynia and hyperalgesia following nerve injury [144], whilst an antagonist of P2X_2/3_ receptor heterodimers reduced sensory abnormalities after CCI [145].

Selective increases in C3, ciliary neurotrophic factor (CNTF) an IL-6-like cytokine and, PTGRF and P2RX2 receptors, in the spinal cord of *Pain & Disability* rats may indicate an exaggerated and unique immune response in these rats, which if restricted to specific locations of the spinal cord, has the potential to activate ascending pathways specific to the expression of disability.

Cellular stress genes: There are also clusters of genes related to the response to cellular stress, which are elevated specifically in *Pain & Disability* rats at day 6, suggesting an exaggerated response to injury. These genes are indicative of the presence of oxidative and cellular stress and include, superoxide dismutase 1 (SOD1) and 2 (SOD2), heme oxygenase (HO), heat shock 10kDa protein (HSP10), apurinic/apyrimidinic endonuclease (APE1), proteasome subunit alpha (PSMA3), metallothionein-1A (MT1A), calpain-1 (CAPN1) and the voltage-dependent anion channel 1 (VDAC1).

Genes regulating cellular processes and homeostasis: Many genes in the cellular structure, cellular signalling, ionic balance and transcription and translation functional groups are identified here to play a role in the expression of disability. For example, mRNA expression levels of the intermediate filament protein, vimentin (VIM), in the lumbar spinal cord are up-regulated at day 2 in rats without persistent disability but then return to normal by day 6, whilst in *Pain & Disability* rats there is a delayed but persistent increase. We have previously shown that VIM, is increased in the midbrain periaqueductal gray of *Pain & Disability* rats [29], indicative of astrocyte proliferation [146]. Hence, a similar pattern of VIM expression occurs at both the spinal and supraspinal levels and is associated with the expression of disability in both cases. The nestin gene (NES) encodes an intermediate filament protein found in both neuronal and glial precursors, and its expression increases in the spinal cord after nerve injury [147]. Here, the mRNA expression pattern of NES, mirrors that of VIM in rats without persistent disability, suggesting cellular proliferation may occur at early stages after CCI in these rats, but normalises by day 6. Therefore, we hypothesise early and transient changes in intermediate filaments NES and VIM in rats without persistent disability aid recovery, but ongoing changes in VIM in *Pain & Disability* rats contributes to the expression of disability, perhaps through an over exaggerated astrocyte proliferation.

### Summary

We have shown that the overall ‘signatures’ of gene regulation changes are different between rats with or without disability. That is, there are more ‘disability-specific’ genes with altered regulation in *Pain & Disability* rats (n = 35), compared to rats without ongoing disability (*Pain alone* and *Pain & Transient Disability*) (n = 12). Of the thirty-five genes regulated in *Pain & Disability* rats, approximately two-thirds (65%) were genes in the neurotransmission and inflammatory and/or cellular stress subgroups (compared to only 34% in rats without disability). Forty percent of the genes regulated in rats without disability (*Pain alone* and *Pain & Transient Disability*) were genes regulating cellular processes and homeostasis (compared to only 6% in *Pain & Disability* rats).

The genes regulated uniquely in rats without persistent disability encode for genes regulating cellular processes and homeostasis, which we suggest contribute to resilience and accelerated recovery from the injury-related triggers of behavioural change. In contrast, select patterns of activation of the neuro-inflammatory repertoire in *Pain & Disability* rats may lead to dramatic alterations in ascending supra-spinal signals.

This view is supported by our earlier experiments, which showed enhanced astrocyte activation and evidence of cell death in lumbar spinal recipient columns of the PAG of *Pain & Disability* rats [28, 29]. It is therefore tempting to suggest these similar midbrain regions receive specific spinal outputs that are regulated selectively by the gene expression changes we have identified in this subgroup of disabled rats.
